# Reported measles cases, measles-related deaths and measles vaccination coverage in Myanmar from 2014 to 2018

**DOI:** 10.1186/s41182-020-0191-4

**Published:** 2020-02-07

**Authors:** Aye Mya Chan Thar, Khin Thet Wai, Anthony D. Harries, Kyaw Lwin Show, Lei Lei Mon, Htar Htar Lin

**Affiliations:** 1 The Expanded Programme on Immunization, Department of Public Health, Ministry of Health and Sports, Naypyitaw, Myanmar; 2 Department of Medical Research, Ministry of Health and Sports, Yangon, Myanmar; 3 International Union Against Tuberculosis and Lung Disease, Paris, France; 4 London School of Hygiene and Tropical Medicine, London, UK; 5 World Health Organization, Yangon, Myanmar

**Keywords:** Myanmar, Measles cases, Measles deaths, Measles vaccination coverage, Measles containing vaccine 1—MCV1, Measles containing vaccine 2—MCV2, SORT IT

## Abstract

**Background:**

There is a global resurgence of measles, consequent upon worldwide stagnating measles vaccination coverage. The study aim was to document trends and characteristics of reported cases of measles, measles-related deaths, and measles vaccination coverage (MCV1—first dose of measles-containing vaccine and MCV2—second dose of measles-containing vaccine) at national and sub-national level in Myanmar over a five year period between 2014 and 2018.

**Methods:**

This was a descriptive study using routine data collected and submitted to the Expanded Programme on Immunization.

**Results:**

Between 2014 and 2018, there were 2673 measles cases of which 2272 (85%) occurred in 2017 and 2018. Five adjacent regions in lower Myanmar were the most affected: in 2017 and 2018, these regions reported 1647 (73%) of the 2272 measles cases in the country. Overall, 73% of measles cases were laboratory confirmed, 21% were epidemiologically linked, and 6% were clinically compatible (clinical diagnosis only), with more laboratory confirmed cases in recent years. Annual measles-related deaths were either zero or one except in 2016 when there were 21 deaths, all occurring in one remote village. In the recent years, the most commonly affected age groups were 0–8 months, 9 months to 4 years, and ≥ 15 years. Among 1907 measles cases with known vaccination status, only 22% had been vaccinated and 70% of those had only received one dose of vaccine. Annual MCV1 coverage nationally varied from 83 to 93% while annual MCV2 coverage nationally varied from 78 to 87%, with no clear trends over the years. Between 2014 and 2018, a high proportion of the 330 townships had MCV coverage < 95%. Over 80% of measles cases were reported from townships that had MCV coverage < 95%.

**Conclusion:**

There have been a large number of measles cases in recent years associated with sub-optimal measles vaccine coverage. Myanmar has already conducted supplemental immunization activities in October and November, 2019. Myanmar also needs to improve routine immunization services and targeted responses to measles outbreaks.

## Introduction

Measles is a highly infectious disease that results from infection with the measles virus. Measles was one of the top causes of childhood morbidity and mortality and responsible for over 2 million childhood deaths each year before the introduction of measles vaccines and the increase in global measles vaccine coverage resulting from the Expanded Programme on Immunization that started in the 1980s [1]. Measles incidence and mortality have declined substantially in the last 20 years due to the improvement in socioeconomic status, better nutrition, and the increasing use of live attenuated measles vaccines administered through routine childhood immunization programmes and mass vaccination campaigns.

Measles vaccination strategies have evolved over time. The World Health Organization (WHO) currently recommends that the first dose of measles-containing vaccine (MCV1) should be administered at 9 months of age in settings with endemic measles. High levels of population immunity necessary to interrupt measles transmission, however, cannot be achieved with a single dose schedule, and a second dose (MCV2) is therefore usually given at 15–18 months of age with a minimum of 4 weeks between the two doses [2].

The WHO reports annually on the number of reported measles cases, the estimated number of deaths, as well as on national measles vaccination coverage. Reported measles cases decreased worldwide from 850,000 to 250,000 between 2000 and 2015 [3]. During the same time period, estimated measles deaths, derived from a model based on reported cases, vaccine coverage, and age-specific fatality ratios [4], decreased by almost 80% [2]. Global measles vaccine coverage with the first dose of MCV1 increased from 72 to 85% from 2000 to 2010, but since then has plateaued at about 85% [3].

In 2012, the World Health Assembly endorsed the Global Vaccine Action Plan with the objective of eliminating measles [5]. The encouraging reductions in measles incidence and mortality have led to five out of six WHO regions adopting the Global Vaccine Action Plan with targets to eliminate measles by 2020 [5]. Measles elimination is defined as the absence of endemic measles virus transmission for ≥ 12 months in a region or other defined geographical area. However, there are significant challenges ahead. In particular, the stagnation of vaccination coverage, for reasons that include under-performing national immunization programmes, hard-to-reach populations, conflict areas, and world-wide anti-vaccine sentiments, has resulted in several severe and protracted measles outbreaks that threaten the achievement of elimination goals [6–8].

Myanmar, a large country in South East Asia, has signed up to eliminate measles by 2023. Before the introduction and scale up of measles vaccine in the country, measles was an important cause of death in infants and young children [9]. Since then, there have been sporadic reports from health facilities about measles-related childhood morbidity and mortality and about decreases in measles admissions and deaths resulting from the measles vaccination programme [10, 11]. There are, however, no recent published studies assessing the annual numbers of reported measles cases and case-fatality rates or the national measles vaccination coverage. Such information would be useful for the country and region as Myanmar strives to interrupt measles transmission and aims to join the four out of the 11 South East Asian countries that have already reached the target of measles elimination.

The aim of this study was to document trends and characteristics of reported cases of measles and measles vaccination coverage in Myanmar over a 5-year period between 2014 and 2018. Specific objectives were to describe at national and sub-national level: first, annual and monthly trends in all reported measles cases (stratified by laboratory-confirmed, epidemiologically linked or clinically compatible) and measles-related deaths; second, demographic and other characteristics of reported measles cases per year, including age, sex, township location, and vaccination history; third, annual trends in reported measles vaccination coverage at the township level with one vaccination only (MCV1) and two vaccinations (MCV1 and MCV2); and fourth, associations between measles cases and MCV coverage in townships.

## Methods

### Study design

This was a descriptive study using routine data which was collected and submitted to the Expanded Programme on Immunization.

### Setting

#### General setting

**Fig. 1:**
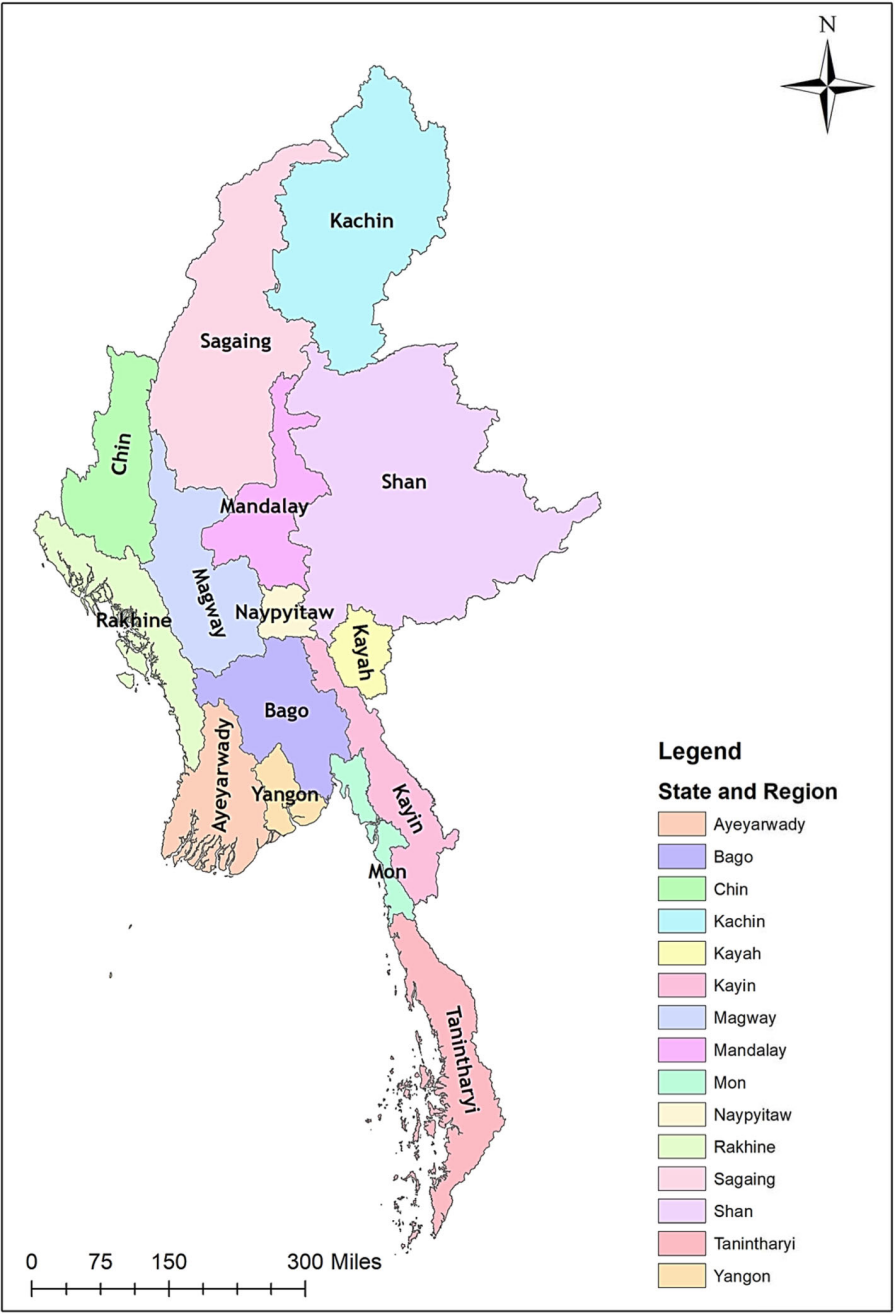
Map of Myanmar showing states and regions

Myanmar is located in the South East Asia Region, bordering the Republic of China in the North/North East, Laos in the East, Thailand in the South East, Bangladesh in the West, and India in the North West. The country is divided administratively into Nay Pyi Taw Union Territory (housing the capital Nay Pyi Taw) and 14 States and Regions (Fig. 1). It consists of 74 Districts, 330 Townships, 398 Towns, 3065 Wards, 13,619 Village Tracts, and 64,134 Villages. The principal geographic features are the delta region and the central plain surrounded by mountains. Myanmar enjoys a tropical climate with three different seasons: wet (June to September), cool (October to January), and hot (February to May). Based on the 2014 national census, the country has a population of nearly 52 million with an urban-rural population ratio of 30:70 [12]. The people live in an area of 676,577 km^2^ with a population density of 76 per km^2^ [12].

#### Study-specific setting

##### Measles surveillance in Myanmar

Myanmar has initiated a case-based measles surveillance system since 2010. Any suspected measles case (defined as any case with fever and maculopapular rash) has to be investigated within 48 h of notification using a standardized case investigation form. This is done by any designated health staff. In all suspected measles cases and within 4–28 days from the onset of the rash, blood has to be collected and the serum sent to one of the proficient laboratories in Yangon or Mandalay. Diagnostic confirmation is done by serological testing for measles specific Ig M antibody using the measles-specific Ig M ELISA Test kit “Enzygnost Anti-Measles Virus/IgM Kit” from Siemens Diagnostics Products, Germany.

Based on the results, any suspected measles case is further classified as follows: (i) laboratory confirmed measles case—suspected measles case with positive serology; (ii) epidemiologically linked measles case—suspected measles case that can be connected geographically and in time to a confirmed measles case; (iii) clinically compatible measles case (clinically diagnosis only)—suspected measles case with cough, coryza, or conjunctivitis in whom there is no adequate laboratory specimen or in whom there is no linkage with any confirmed index measles case; and (iv) discarded case—suspected measles case with a negative serological result [13]. These categories are mutually exclusive. Any measles case which is laboratory confirmed, epidemiologically linked, or clinically compatible with measles is documented and recorded as a “reported measles case” to the health system [13]. A measles-related death is any death occurring within 30 days of the onset of rash in a “reported measles case.”

Case investigation forms are sent to the Central Epidemiology Unit (CEU) and the Expanded Programme on Immunization (EPI) through the township and state/region public health department. The classification of measles cases is done by the CEU, EPI, and WHO so that each case can be placed in one of the four categories described earlier. The data from these forms are entered into an electronic EXCEL file in CEU which is then sent to WHO Country office and used by WHO and EPI for surveillance and response. There is limited reporting of measles from the private sector.

##### Measles vaccination and data reporting systems

Measles vaccination is carried out as follows. EPI coordinates the administration of the immunization programme. All infants are vaccinated with the first dose of measles-rubella vaccine at 9 months of age (MCV1), unless there are contraindications such as the acquired immune deficiency syndrome (AIDS). The second dose of measles-rubella vaccine is given at 18 months of age (MCV2). Midwives provide these routine vaccinations at the health facility and at the community level through outreach sessions. A mass vaccination campaign was also implemented in 2015 targeting children from 9 months to 15 years as part of a WHO/national recommendation to boost vaccination coverage [2]. The goal is to achieve ≥ 95% measles vaccine coverage at the township level in order to interrupt measles transmission and eliminate measles [2].

Monthly vaccination data are recorded at the sub-rural health center level and reported to the rural health centres (RHC) by midwives in the last week of every month and data are then collated at the RHC. The RHC data are reported upwards to township public health departments in the last week of the month and again collated at the township level. The township data are then entered into the web-based District Health Information System (DHIS 2) during first week of the following month. The DHIS 2 was rolled out throughout the country in 2017but before this a paper-based record was used to report to the Health Management Information System (HMIS) and EPI.

Measles vaccination coverage requires a numerator and denominator. MCV1 coverage is defined as the number of children < 1 year of age given the first dose of measles vaccination divided by number of children < 1 year of age. MCV2 coverage is number of children < 2 years of age given the second dose of measles vaccination divided by number of children < 1 year of age. The denominator is acquired through annual head counting which is carried out at the end of each year. Annual head counting (number of persons in the catchment area stratified by age group: < 1; < 5; < 15; women of child-bearing age) is carried out at sub-rural health center level usually by midwives and the data are then collated at the RHC and township level to obtain township level population data [14]. A growth rate is applied to this number to calculate a projected population that is targeted the following year for vaccination services. These data are transmitted upwards to state, regional, and national level [14]. The central unit of EPI then calculates the national population data. For example, the projected number of children < 1 year of age for 2018 was 0.95 million nationally. Data on measles vaccination coverage is then made available on HMIS and EPI and collated in an annual EXCEL file which is based in EPI.

### Study population

Persons with reported measles in Myanmar and infants and children immunized with measles vaccine (MCV1 and MCV2) between 2014 and 2018 were included in the study.

### Data variables, sources of data, and data collection

Data variables included: individual numbers of “reported measles cases” by month and by year, stratified by those that were laboratory confirmed, epidemiologically linked, and clinically compatible; individual numbers of measles-related deaths by year; age, sex, township location, and previous measles vaccination history of measles cases; national annual MCV1 and MCV2 coverage; and townships achieving ≥ 95% coverage of MCV1 and MCV2.

The sources of data were two separate EXCEL files available in EPI for measles cases and for measles vaccination coverage. Data were collected for the current study between March and July 2019 into an EXCEL spread sheet.

### Analysis and statistics

Data were exported to and analyzed using EpiData (version 2.2.2.182 for analysis, EpiData Association, Odense, Denmark). A descriptive analysis was performed using frequencies and proportions for reported measles cases and measles-related deaths. Measles cases were also analyzed in relation to certain characteristics such as age group, gender, urban/rural residence, and history of previous vaccination. A descriptive analysis was also carried out for measles vaccination coverage (MCV1 and MCV2) over the 5-year period at national and township level. Finally, reported measles cases were compared over the 5 years between townships with ≥ 95% and < 95% vaccination coverage. Nationwide maps of measles cases across 5 years (2014–2018) were generated through a geographical information system using Arc GIS software (version; ArcGIS Desktop 10.6.1 Esri Inc. Ca).

## Results

### Annual and monthly trends in reported measles cases and measles deaths

**Fig. 2:**
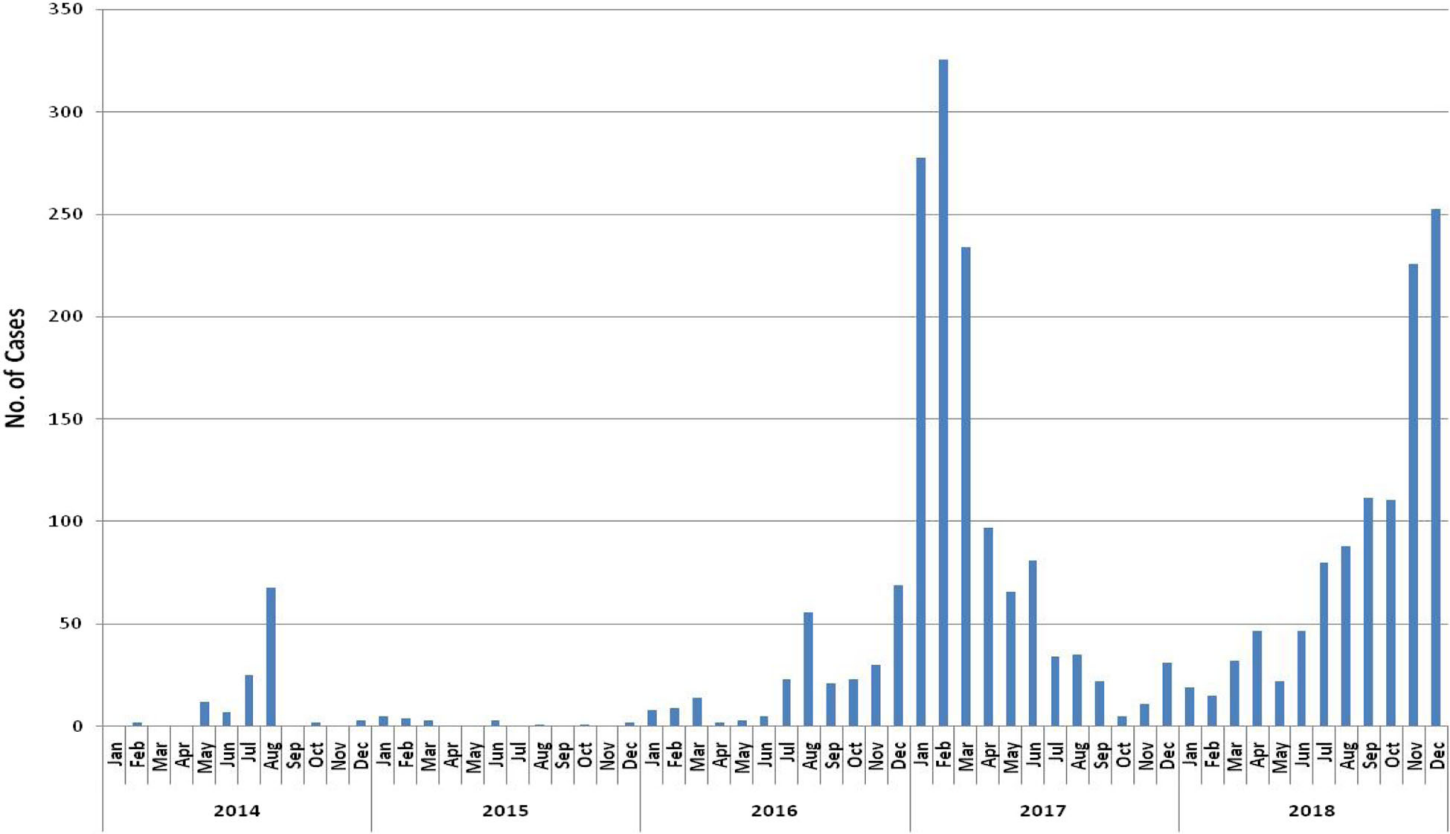
Annual and monthly trends of all reported measles cases in Myanmar: 2014–2018

Annual and monthly trends in reported measles cases between January 2014 and December 2018 are shown in Fig. 2. There was a total of 2673 cases of which 2272 (85%) occurred in 2017 and 2018. In those last 2 years, monthly numbers of measles cases exceeded 200 in January, February, and March 2017 and in November and December 2018.

**Fig. 3:**
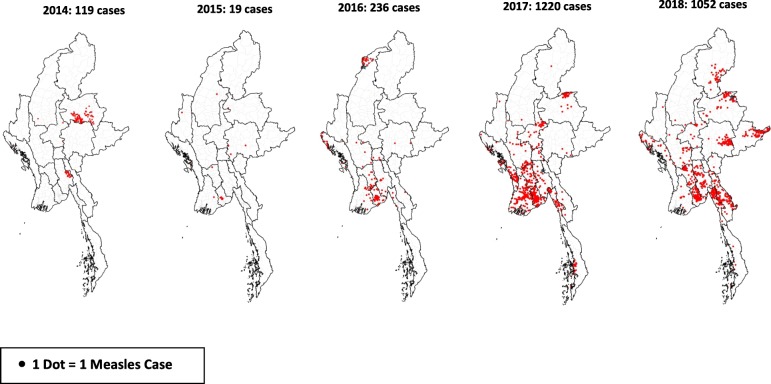
Geospatial distribution of reported measles cases in Myanmar: 2014–2018

**Fig. 4:**
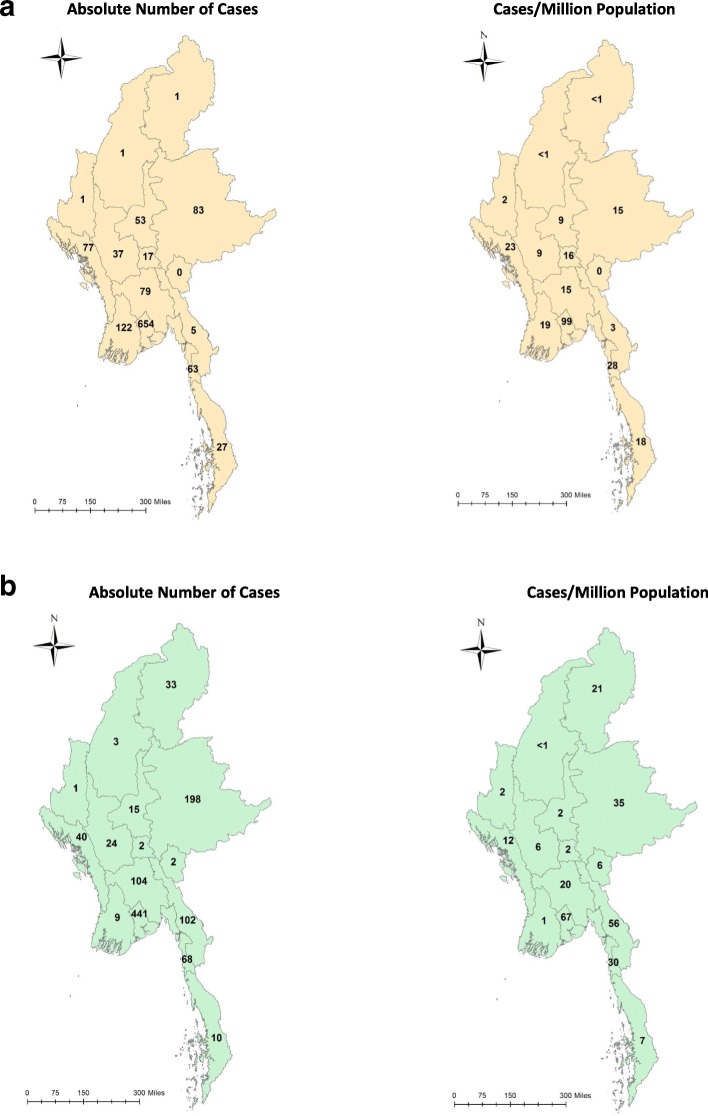
Geospatial distribution of reported measles cases in Myanmar in 2017 and 2018. **a** Absolute number of cases and number of cases per million population in 2017. **b** Absolute number of cases and number of cases per million population in 2018

Figure 3 shows the geospatial distribution of individual measles cases during this period while Fig. 4 shows the absolute number of measles cases and the number of measles cases per million population in each State/region of the country for 2017 (Fig. 4a) and 2018 (Fig. 4b). Ayeyarwady, Yangon, Bago, Mon, and Kayin in lower Myanmar, i.e., the delta and lowlands, were the main regions affected: in 2017 and 2018, these five regions reported 1647 (73%) measles cases out of 2272 national cases. The other two regions in eastern part of Myanmar in the hills and western part of Myanmar in the coastal region were Shan and Rakhine state which in 2017 and 2018 reported 398 (18%) measles cases.

Annual reported measles cases (classified into those that were laboratory confirmed, epidemiologically linked and clinically compatible) and annual measles deaths between 2014 and 2018 are shown in Table 1. Over the whole 5-year period, 73% of measles cases were laboratory confirmed, 21% were epidemiologically linked, and the remainder clinically compatible. In 2014 and 2015, less than one-third of measles cases were laboratory confirmed, but the proportion with laboratory confirmation strikingly increased from 2016 onwards to 70% and above for each of the 3 years. Most of the other measles cases were epidemiologically linked. Annual measles-related deaths were either zero or one in the 5-year period except for 2016 when there were 21 deaths (16 children and 5 adults): these deaths, none of which were laboratory-confirmed, all occurred in one hard-to-reach remote village in Naga self-administered region: the cause of death was ascertained by an investigating team using verbal autopsy where it was concluded that only three deaths could confidently be attributed to measles.

**Table 1:** Annual reported measles cases, stratified by laboratory confirmed, epidemiologically linked, and clinically compatible and annual reported measles-related deaths in Myanmar: 2014–2018

Characteristics	2014	2015	2016	2017	2018	Total
	*n* (%)	*n* (%)	*n* (%)	*n* (%)	*n* (%)	*n* (%)
All measles cases	119	19	263	1220	1052	2673
Classification:						
Laboratory confirmed	24 (20)	6 (32)	194 (74)	990 (81)	741 (70)	1955 (73)
Epidemiologically linked	93 (78)	0 (0)	45 (17)	145 (12)	269 (26)	552 (21)
Clinically compatible	2 (2)	13 (68)	24 (9)	85 (7)	42 (4)	166 (6)
All measles-related deaths	0 (0)	0 (0)	21 (8)	1 (< 1)	1 (< 1)	23 (1)

Percentages are column percentages

Laboratory confirmed = suspected measles case with positive serology

Epidemiologically linked = suspected measles case connected geographically and in time to a confirmed measles case

Clinically compatible = suspected measles case with no adequate laboratory specimen or in whom there is no linkage with any confirmed measles case

### Demographic and other characteristics of reported measles cases per year

Demographic and other characteristics of measles cases per year from 2014 to 2018 are shown in Table 2. In 2014, measles was most commonly reported in children in the age group 5–14 years. From 2015 onwards, three age groups became more common: infants < 9 months of age, young children 9 months to 4 years of age, and adolescents and adults aged ≥15 years. Females were more commonly reported with measles compared with males except for 2017 when more males were reported. Overall, there were more reported measles cases from urban compared with rural areas, but this ratio varied from year to year. Among 1907 measles cases with known vaccination status, only 22% had been vaccinated and 70% of those had only received one dose of vaccine: between 2014 and 2016, all those who were vaccinated had received only one dose.

**Table 2:** Demographic and vaccination characteristics of annual reported measles cases in Myanmar: 2014–2018

Characteristics	2014	2015	2016	2017	2018	Total
	*n* (%)	*n* (%)	*n* (%)	*n* (%)	*n* (%)	*n* (%)
All measles cases	119 (100)	19 (100)	263 (100)	1220 (100)	1052 (100)	2673 (100)
Age group						
0–8 months	3 (2)	0 (0)	26 (10)	114 (9)	54 (5)	197 (7)
9 months to 4 years	15 (13)	5 (26)	90 (34)	440 (36)	338 (32)	888 (33)
5–9 years	39 (33)	6 (32)	28 (11)	123 (10)	271 (26)	467 (17)
10–14 years	45 (38)	1 (5)	12 (5)	32 (3)	110 (10)	200 (8)
≥ 15 years	17 (14)	7 (37)	98 (37)	509 (42)	279 (27)	910 (35)
Data unavailable	0 (0)	0 (0)	9 (3)	2 (< 1)	0 (0)	11 (< 1)
Gender						
Male	15 (13)	7 (37)	123 (47)	638 (52)	498 (47)	1281 (48)
Female	104 (87)	12 (63)	140 (53)	582 (48)	554 (53)	1392 (52)
Residence						
Urban	72 (61)	9 (47)	122 (46)	697 (57)	451 (43)	1351 (51)
Rural	47 (39)	9 (47)	124 (47)	423 (35)	506 (48)	1109 (41)
Data unavailable	0 (0)	1 (5)	17 (7)	100 (8)	95 (9)	213 (8)
Previous measles vaccine						
Yes	4 (3)	11 (58)	58 (22)	215 (17)	124 (12)	412 (15)
No	8 (7)	3 (16)	170 (65)	775 (64)	539 (51)	1495 (56)
Data unavailable	107 (90)	5 (26)	35 (13)	230 (19)	389 (37)	766 (29)
Number vaccine doses^a^						
1 dose	4 (100)	11 (100)	58 (100)	126 (59)	89 (72)	288 (70)
2 doses	0 (0)	0 (0)	0 (0)	80 (37)	27 (22)	107 (26)
> 2 doses	0 (0)	0 (0)	0 (0)	9 (4)	8 (6)	17 (4)

Percentages are column percentages

^a^Denominator = number of persons with previous measles vaccination

### Annual trends in reported measles vaccination coverage at national and township level

Annual trends in measles vaccination at the national level and in the 330 townships of the country between 2014 and 2018 are shown in Table 3. Annual coverage of MCV1 nationally varied from 83 to 93% while that of MCV2 varied from 78 to 87%, with no clear trend seen over the 5-year period in either case. Similarly, the proportion of townships that had annual MCV1 coverage ≥ 95% varied from 4% in 2017 to 59% in 2018 while the proportion of townships that had annual MCV2 coverage ≥ 95% varied from 4% in 2017 to 28% in 2018.

**Table 3:** Measles vaccination coverage in Myanmar: 2014–2018

A: Measles vaccination coverage at national level					
Characteristics	2014	2015	2016	2017	2018
	%	%	%	%	%
Coverage of MCV1	88	84	91	83	93
Coverage of MCV2	82	78	86	80	87
B: Measles vaccination coverage at township level					
Characteristics	2014	2015	2016	2017	2018
	*n* (%)	*n* (%)	*n* (%)	*n* (%)	*n* (%)
Townships where MCV1 ≥ 95%	90 (27)	63 (19)	130 (39)	13 (4)	194 (59)
Townships where MCV2 ≥ 95%	31 (9)	26 (8)	53 (16)	14 (4)	94 (28)

Total number of townships in Myanmar = 330

MCV1 = first dose of measles containing vaccine

MCV2 = second dose of measles containing vaccine

### Associations between measles cases and MCV coverage at the township level

Associations between measles cases and MCV coverage (MCV1 and MCV2) at the township level are shown in Table 4. Over the whole 5-year period, 80% of measles cases occurred in townships with MCV1 coverage < 95% and 88% of measles cases occurred in townships with MCV2 coverage < 95%. These proportions varied each year with no clear trends shown.

**Table 4:** Associations between annual reported measles cases and MCV coverage at the township level, Myanmar: 2014 to 2018

A: Measles cases in relation to MCV1 Coverage			
Year	All measles cases	Measles cases in townships with MCV1 coverage ≥ 95%	Measles cases in townships with MCV1 coverage < 95%
	*n*	*n* (%)	*n* (%)
2014	119	1 (0.8)	118 (99.2)
2015	19	4 (21.1)	15 (78.9)
2016	263	79 (30.0)	184 (70.0)
2017	1220	11 (0.9)	1209 (99.1)
2018	1052	451 (42.9)	601 (57.1)
B: Measles cases in relation to MCV2 Coverage			
Year	All measles cases	Measles cases in townships with MCV2 coverage ≥ 95%	Measles cases in townships with MCV2 coverage < 95%
	*n*	*n* (%)	*n* (%)
2014	119	28 (23.5)	91 (76.5)
2015	19	1 (5.3)	18 (94.7)
2016	263	33 (12.5)	230 (87.5)
2017	1220	20 (1.6)	1200 (98.4)
2018	1052	247 (23.5)	805 (76.5)

MCV1 = first dose of measles containing vaccine

MCV2 = second dose of measles containing vaccine

## Discussion

This is the first national study to report on measles cases and deaths in Myanmar over a 5-year period (2014–2018), the epidemiological profile of the disease, and measles vaccination coverage. Measles cases have increased dramatically in the last 2 years and this appears to be related to poor measles vaccination coverage.

### Key study findings

First, there was a dramatic upsurge in reported measles cases in the country in 2017 and 2018. During this 2-year period, the disease was most concentrated in five adjacent regions in the delta and lowlands and one region each in the hills and in the coastal area. There was no specific seasonal correlation and high numbers of measles cases occurred in both the cool and the hot seasons. It is likely from our data that in the last 3 years, these were true measles cases because most were confirmed in the laboratory and the remainder were epidemiologically linked. This contrasts with the earlier period in 2014 and 2015 when less than one-third of cases in Myanmar were laboratory confirmed. This strong data on confirmed measles cases in Myanmar also contrasts with epidemiological reports from two African countries where laboratory confirmation of measles cases was less than 50% in Senegal and less than 25% in the Central African Republic [15, 16].

While measles cases appear to have increased hugely in the first 7 months of 2019 in the African, Western Pacific, and Eastern Mediterranean regions, the South East Asia region and the region of the Americas have seen a 15% decrease in reported cases [17]. Unfortunately, preliminary country-wide data for Myanmar up to August 2019 shows a continuing increase in reported measles cases [17].

Second, there was either zero or one reported death in each of the years apart from 2016 where 21 deaths were recorded in a small remote village. A review of these deaths the following year found that while there had indeed been a severe measles outbreak consequent upon low vaccine coverage in the village, only three of the deaths could confidently be attributed to measles [18]. Nevertheless, undernutrition, overcrowding, and poor access to care in developing countries may be associated with measles mortality rates as high as 1% to 15% [19], and since the start of 2019 in the Philippines, 136 measles deaths have been recorded in the deadliest recent outbreak [20].

Third, the profile of measles cases in the country changed from being largely a disease seen in children aged 5–14 years in 2014–2015 to a disease seen in older age groups and also infants. This age group profile in recent years was very similar to that reported from Senegal [15] and from an outbreak in Sri Lanka [21]. Of interest was the increase in reported measles in infants < 9 months of age, who traditionally receive protection through maternal antibodies in utero and through breast feeding. A report from Ireland indicated a high number of measles cases in children aged 0–5 months and 6–11 months [22], suggesting a waning of immunity in expectant and breast-feeding mothers due to poor uptake of measles immunization.

With respect to previous measles vaccination history, there was missing data in nearly one-third of measles cases. However, where this data was available the majority of cases had not undergone vaccination and in those who had been vaccinated, most had only received one dose, similar findings to those reported from Sri Lanka [21]. Nevertheless, it was reported that a small number of measles cases had received two or more doses of vaccine. The accuracy of these reports needs to be checked. However, measles vaccine failures have recently been reported from China [23, 24]. The reasons are not clear and further research is needed to better understand this phenomenon.

Fourth, MCV coverage did not reach standards required to interrupt measles transmission. In 2010, the World Health Assembly set three milestones for measles control, one of which was to increase MCV1 coverage to ≥ 90% at the national level [25]. This milestone was achieved only twice in the 5 years, in 2016 and 2018. MCV2 coverage never reached 90% in all 5 years. In 2017, WHO recommended ≥ 95% measles vaccine coverage at the district level [2], and this was adopted as the township target level for Myanmar. This target was achieved for MCV1 in 59% of townships in 2018 and was achieved for MCV2 in 28% of townships in the same year. The nadir was in 2017 when less than 5% of townships achieved the 95% goal for MCV1 and MCV2.

Finally, our study findings also clearly showed that measles was more common in townships with < 95% MCV 1 and MCV 2 coverage indicating the importance of vaccination coverage for individual protection and herd immunity. This phenomenon has been well demonstrated in the recent measles outbreaks in the European Union in 2017 and 2018 [26].

### Strengths and limitations of the study

The strengths of this study were the good surveillance data over 5 years at national and sub-national level on measles cases (the majority with laboratory confirmation), measles deaths, and vaccination coverage. The reporting of the study was also in line with the Strengthening the Reporting of Observational Studies in Epidemiology (STROBE) [27]. Limitations related to the missing data about vaccination coverage in reported measles cases, and the lack of information about the burden of measles cases and deaths which are not reported.

### Programmatic implications

Measures such as outbreak response immunization activities are being undertaken to contain these measles outbreaks in Myanmar that have occurred in the last 2 years and are continuing to increase [17]. Measles is the classic example of a vaccine preventable disease and live-attenuated measles vaccines are among the most highly effective vaccines available with a proven safety record [28]. Among those properly vaccinated with two doses, immunity is probably life-long [29].

Myanmar has conducted supplemental immunization activities (SIAs) in October and November 2019. In October, 96 townships with overall low vaccination coverage were visited and all children between 9 months and 5 years and 6 months, regardless of vaccination status, received one dose of measles-containing vaccine. In November, the remaining townships were visited and the children similarly vaccinated. Inadvertently giving an extra dose of vaccine to someone already previously vaccinated is safe and does not appear to cause any adverse health effects [30]. The national immunization programme will simultaneously improve implementation of routine childhood immunization services for MCV1 and MCV2 by having a fully functioning supply chain mechanism and reducing vaccine wastage to as low as possible as recommended in the Global Vaccine Action Plan [5]. Finally, targeted responses to settings where a measles outbreak is occurring need to be more efficient so that timely post-exposure prophylaxis can be administered if necessary. This approach has been successful in high and low-middle-income countries in containing isolated measles outbreaks [31, 32].

Renewed efforts are needed to bring measles back under control. Key research areas for moving forward include affordable and point-of-care diagnostic tools, effective strategies to increase coverage of MCV1 and MCV2, and better engagement with communities to plan, implement, and monitor health services including vaccinations [33]. There is a need to move quickly in these areas before measles becomes a national public health emergency.

## Conclusion

This study has described reported measles cases and deaths and measles vaccination coverage in Myanmar over a 5-year period between 2014 and 2018. During this period, there were 2673 cases of which 85% occurred in the last 2 years. There were 23 measles deaths, 21 of which occurred in 2016 in one village. In the five years. MCV1 coverage nationally varied from 83 to 93% while that of MCV2 coverage varied from 78 to 87%. Similarly, the proportion of townships with MCV1 coverage ≥ 95% varied from 4 to 59% while townships with MCV2 coverage ≥ 95% varied from 4 to 28%. More measles cases were reported from townships with < 95% measles vaccination coverage. The plans that Myanmar has to improve measles vaccination coverage are discussed.

## Data Availability

All data generated or analyzed during this study are included in this published article.

## Abbreviations

CEU: Central Epidemiology Unit
DHIS 2: District Health Information System
EPI: Expanded Programme on Immunization
HMIS: Health Management Information System
MCV1: Measles-containing vaccine (1st dose)
MCV2: Measles-containing vaccine (2nd dose)
RHC: Rural Health Centre
STROBE: Strengthening the Reporting of Observational Studies in Epidemiology
